# Low frequencies in the display vocalization of the Western Capercaillie (*Tetrao urogallus*)

**DOI:** 10.7717/peerj.9189

**Published:** 2020-07-08

**Authors:** Vlastimil Hart, Richard Policht, Vojtěch Jandák, Marek Brothánek, Hynek Burda

**Affiliations:** 1 Department of Game Management and Wildlife Biology, Faculty of Forestry and Wood Sciences, Czech University of Life Sciences Prague, Praha, Czech Republic; 2 Faculty of Electrical Engineering, Czech Technical University in Prague, Prague, Czech Republic

**Keywords:** Communication, Vocal individuality, Grouse, Acoustic communication, Low-frequency signals, Courtship behaviour

## Abstract

Only a few bird species are known to produce low-frequency vocalizations. We analyzed the display vocalizations of Western Capercaillie males kept in breeding centers and identified harmonically structured signals with a fundamental frequency of 28.7 ± 1.2 Hz (25.6–31.6 Hz). These low-frequency components temporally overlap with the Whetting phase (96% of its duration) and they significantly contribute to the distinct vocal expression between individuals. The resulting model of discrimination analysis classified 67.6% vocalizations (63%, cross-validated result) correctly to the specific individual in comparison to the probability by chance of 12.5%. We discuss a possible function of low-frequency components that remains unclear. The occurrence of such low frequencies is surprising as this grouse is substantially smaller than cassowaries (Southern cassowary *Casuarius casuarius* and Dwarf cassowary *Casuarius bennetti*) , the species that produces similarly low frequencies. Because these low frequency components temporarily overlap with the Whetting phase, they are hardly audible from a distance larger than several meters.

## Introduction

While the uses of sound occurring in the range of human hearing have been intensively studied, and similarly studied sounds above the upper limit of human hearing (e.g., ultrasounds in bats, toothed whales, rodents, frogs and insects), very little is known about the sound below that range. Such frequencies (infrasound) were documented in whales (Berta, Sumich & Kovacs, 2006; Mourlam & Orliac, 2017), elephants (Larom et al., 1997; McComb et al., 2000; McComb et al., 2003), tigers (Von Muggenthaler, 2000), rhinoceroses (Policht et al., 2008; Von Muggenthaler et al., 2003). Only a few bird species are known to produce very low frequencies. Cassowaries produce booming calls with a minimum frequency down to 32 Hz in the Southern Cassowary (*Casuarius casuarius*) and 23 Hz in the Dwarf Cassowary (*Casuarius bennetti*) (Mack & Jones, 2003). Boom calls of the Great Curassows (*Crax rubra*) have frequencies of maximum amplitude between 100 and 150 Hz (Baldo & Mennill, 2011), booms of the Houbara bustard (*Chlamydotis undulata*) with fundamental frequency ranging between 40 Hz and 54 Hz (Cornec, Hingrat & Rybak, 2014), those of the Eurasian bittern (*Botaurus stellaris*) ranging from 86.6 Hz to 248.5 Hz (Puglisi et al., 2001). Corncrakes are known to produce low-frequency soft calls which largely overlap with the frequency spectrum of the background noise (Rek, 2013; Rek & Osiejuk, 2011). Various versions of low-frequency signals also are produced by doves, e.g., the song of the tambourine dove reaches a minimum frequency of 280 Hz (Osiejuk et al., 2019) or in the spotted dove 524 ± 36 Hz (mean ± SD) (Guo, Bonebrake & Dingle, 2016). All these calls are low-frequency calls, yet they do not fall into the real infrasound range (with frequencies below 20 Hz). The appearance of these low-frequency signals was especially observed in non-passerines of larger body size. As a matter of general bio-acoustic principle, larger animals including birds tend to produce calls of lower frequencies than smaller ones (Fletcher, 2005; Wallschläger, 1980). The validity of this rule has been found not only in songs (Azar & Bell, 2016; Brenowitz, 1982; Ryan & Brenowitz, 1985) but in calls as well (Azar & Bell, 2016; Billings, 2018; Martin et al., 2011). In addition to the influence of body size on the vocalization of grouse, other morphological adaptations can affect the resulting design of produced signals. Grouse (Tetraonidae) have an enlarged oesophagus which inflates like a balloon during mating vocalizations (Moss & Lockie, 1979) and functions as resonating chamber to amplify their signals (Ziswiler & Farner, 1972).

Previous work by Lieser, Berthold & Manley (2005) did not succeed in finding any of the low-frequency components described by Moss & Lockie (1979) based on the vocalizations of only one male. Moss & Lockie (1979) recorded signal components below 40 Hz but Lieser, Berthold & Manley (2005) did not notice any signal below 100 Hz in the vocalization of both wild and captive birds. In such fact, the existence of low-frequency components remains controversial. We therefore tested whether the Western Capercaillie with an average weight of 4–6 kg can really produce any low-frequency signals. If so, we could expect that some frequency parameters could serve as an honest signal of male body mass and/or condition, especially a negative relationship between acoustic parameters and body mass explained by correlation with the length of the trachea, size of the syrinx and vocal track resonance (Lambrechts, 1996; Wallschläger, 1980).

In that case, we could expect individual differences in vocalization among different males. We analysed recordings of vocalization of birds kept in breeding centres in the Czech Republic, Poland and Bavaria in order (1) to describe the acoustical features of potential low-frequency components, and (2) to test the hypothesis that such components have the possibility to encode individual identity.

## Materials & Methods

### Ethics statement

The research was conducted in accordance with the guidelines of the Animal Behaviour Society for the ethical use of animals in research. The study (recording of acoustic signals of semi-tame male capercaillies held in breeding stations) was not invasive and the animals were not handled, disturbed, or manipulated; thus it was not considered an experiment according to the Guide for Care and Use of Animals of the Czech University of Life Sciences Prague and the laws of the Czech Republic. The breeding centre staff was responsible for all bird husbandry and care (permission nu. S/04177/BE/14).

### Study objects and locations

We analysed recordings of vocalization of altogether eight adult males of the Western Capercaillie (*Tetrao urogallus*) kept in breeding centres in the Czech Republic (Šumava), Poland (Wisła Forestry District in Jaworzynka), and Germany (Bayerwald-Tierpark Lohberg). All the subjects were kept in captivity and were housed either in pairs or with several females. Their vocalizations were recorded from March 3 to May 5, 2016. This period coincided with the courting season and all these captive birds displayed courtship behaviour.

### Recording and acoustic analyses

For recording, we used Olympus Linear PCM LS-5 and ZOOM H5 recorders with Sennheiser ME 62 omnidirectional microphone (frequency response 20 Hz–20 kHz) with K6 powering module and QTC50 microphone (Earthworks Inc. Milford, NH, USA) (frequency response 3 Hz–50 kHz) using sampling rate 44.1 kHz and 16 bit sample size. We were using two equipment sets because we needed to record neighbouring males synchronously in order to be sure which songs belonged to the correct individual. When we were recording successively, we were not sure of identity of the caller when neighbouring males were calling at the same time. Therefore we gave a microphone to each neighbouring male and recorded simultaneously.

**Figure 1:**
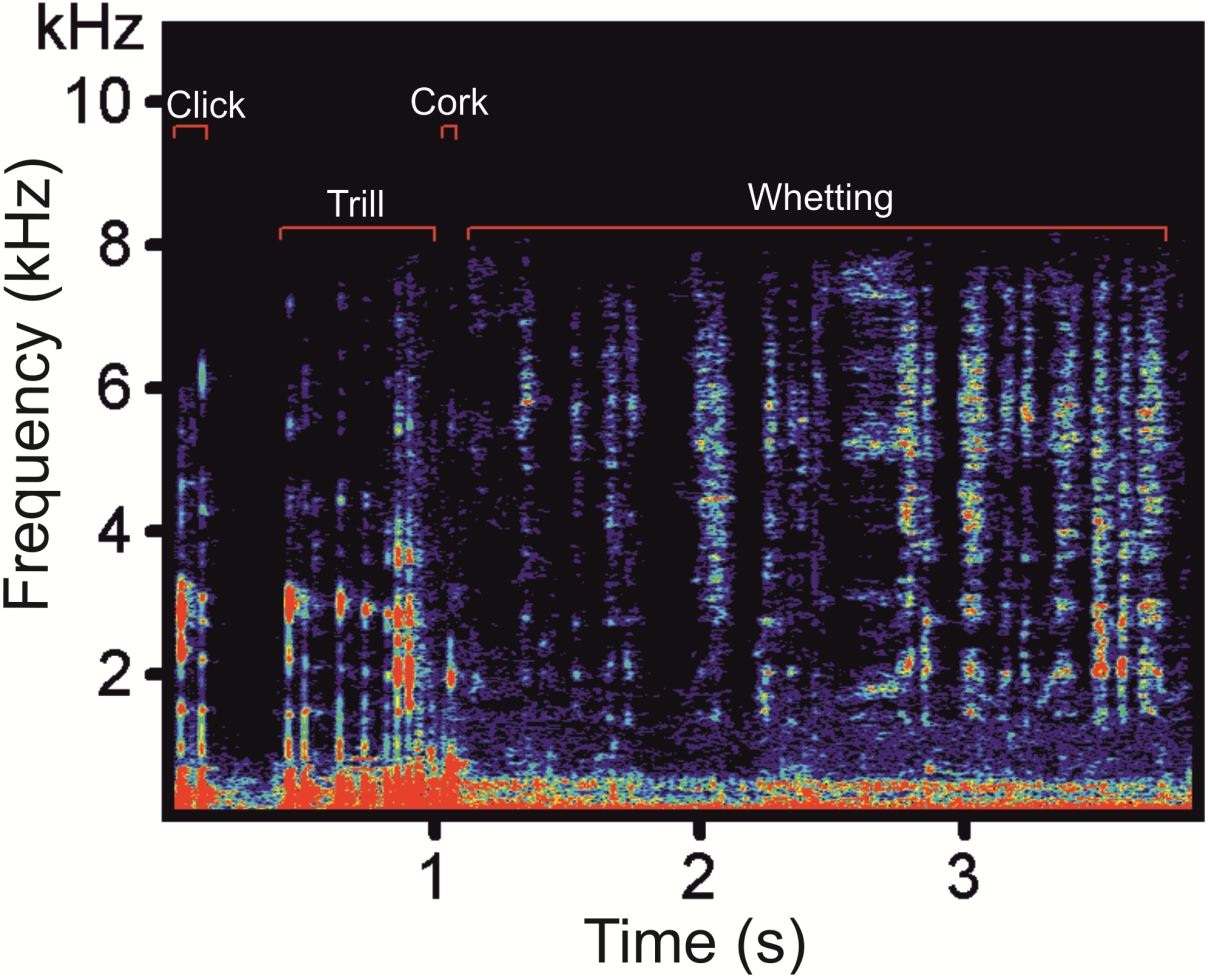
A spectrogram of the Western Capercaillie display vocalization showing the four typical phases.

Prior to analysis, we down-sampled a sampling rate at 22.05 kHz. The distance between the subjects and the microphone ranged between 0.5 and 3 m. We selected vocalizations which were clear and not overlapped by any disturbing noise including vocalizations of other males. Altogether, we analysed 108 display vocalizations (62 vocalizations of four males from the Czech Republic, 32 vocalizations of three males from Poland, and 14 vocalizations of one male from Germany). In the selected display vocalizations, we manually labelled vocal phases (Clicks, Trill, Cork and Whetting, see Fig. 1) and saved into spectrograms using Avisoft-SASLab Pro version 5.2. (Specht, 2016). We created two types of spectrograms: (1) spectrograms with high temporal resolution (FFT length 1024, frame size 100%, overlap 87.5%, Hamming window, time resolution 5.8 ms, frequency resolution 22 Hz, sampling rate 22.05 kHz); and (2) spectrograms with high-frequency resolution in which we decreased the sampling rate to 4 kHz in order to increase the frequency resolution to 4 Hz (FFT length 1024, frame size 100%, overlap 93.75%, Hamming window, time resolution 32 ms) allowing better inspection of very low-frequency signal components (Policht et al., 2008). Frequency resolution increases with decreased sampling frequency (Avisoft Bioacoustics, R. Specht, Berlin, Germany). In spectrograms with high temporal resolution, we measured temporal parameters of the whole main phases, the Whetting and the Trill, while in spectrograms with high-frequency resolution we measured parameters of the low-frequency components. Frequency parameters were measured from the amplitude spectrum (linear). We labelled these signals on spectrograms for calculation of their duration and identified the fundamental frequency and upper harmonics. We measured the following acoustic parameters: song duration (Dur), fundamental frequency (F0), frequency of the highest intensity (Peak F), duration of the low-frequency component (LowF dur), duration of the Trill phase (Trill dur) and Whetting duration (Whetting dur). Additionally we computed temporal overlaps of LowF dur and Trill dur (LowF to Trill overlap) and Whetting dur (LowF to Whetting overlap).

### Calibration of recording equipment

The recording sets were tested for sensitivity to low-frequency signals. We tested the following equipment combinations: Set 1 (ZOOM H5 recorder and QTC50 microphone), and Set 2 (Olympus Linear PCM LS-5 recorder and Sennheiser ME 62 omnidirectional microphone). The tests were conducted in the anechoic room at the Faculty of Electrical Engineering, Czech Technical University in Prague. The room provides an acoustic free field and sufficient signal-to-noise ratio across the whole frequency range of interest (15 Hz–20 kHz). The measurement system Brüel & Kjær Pulse type 3109 was used for the measurement and analyses of signals recorded by the equipment under tests. Pure tones were used as measurement signals. In the frequency range from 15 Hz up to 45 Hz, the fine frequency step of 2 Hz was used. In the frequency range from 45 Hz to 20 kHz, center frequencies of octave bands were used. Pure tones were played successively. For the calibration we used a high precession microphone Brüel & Kjær type 4190 as the reference one. All microphones were placed at a 1m distance from the sound source (12-inch active subwoofer with 2 way satellite loudspeaker box) producing the test signals generated by the Brüel & Kjær Pulse system. All signals were recorded and then post processed by the Brüel & Kjær Pulse system using one-twelfth octave band analyse. In general, the frequency range of equipment is defined by decrease of the frequency response of 10 dB. As the result, it was found that the frequency response of Set 1 is flat in the frequency range of interest (decrease of 3 dB at 15 Hz), which fully corresponds to the nominal values declared by the manufacturers. The frequency range of Set 2 was determined from 20 Hz up to 20 kHz. The same frequency range is declared by the manufacturers. Both recording sets are suitable for recording the described low-frequency components of vocalizations of the Western Capercaillie males.

### Statistical analysis

We used stepwise discriminant-function analysis (DFA) giving a set of discriminant functions combining the parameters of the low-frequency components per individual birds in a way that the signals are assigned to the individuals and thus give a percentage of correctly classified low-frequency signals (Cornec, Hingrat & Rybak, 2014). We excluded highly inter-correlated parameters with *r* > 0.77 (McGarigal, Cushman & Stafford, 2000). Data entered into the DFA were Z –standardized (mean subtracted from each value and divided by standard deviation).

A priori probabilities of classification were weighted by the number of the calls per individual. We used the leave-one-out cross-validation procedure to validate the results of DFA, where each call was classified by the function derived from all cases but that particular case (see IBM SPSS Statistics 20). Descriptive statistics presents the means ± SD.

## Results

The typical display vocalization of the Western Capercaillie contains four phases: Clicks, Trill, Cork and Whetting (Fig. 1). A series of Clicks represent the most frequent sound produced during courting. These vocalizations are frequently produced both alone (without other remaining phase) and as an introductory part of the full display vocalization. In the latter case, the series of Clicks culminates into the Trill which is formed by a quicker repetition of Clicks. The Trill is immediately followed by the Cork, sounding like pulling the cork up of the bottle. The last phase, Whetting, is formed by syllables sounding like a scraping noise. This harsh, repetitive noisy sound is a long phase with a mean duration of 2.85 ± 0.15s (2.37–3.28).

**Figure 2:**
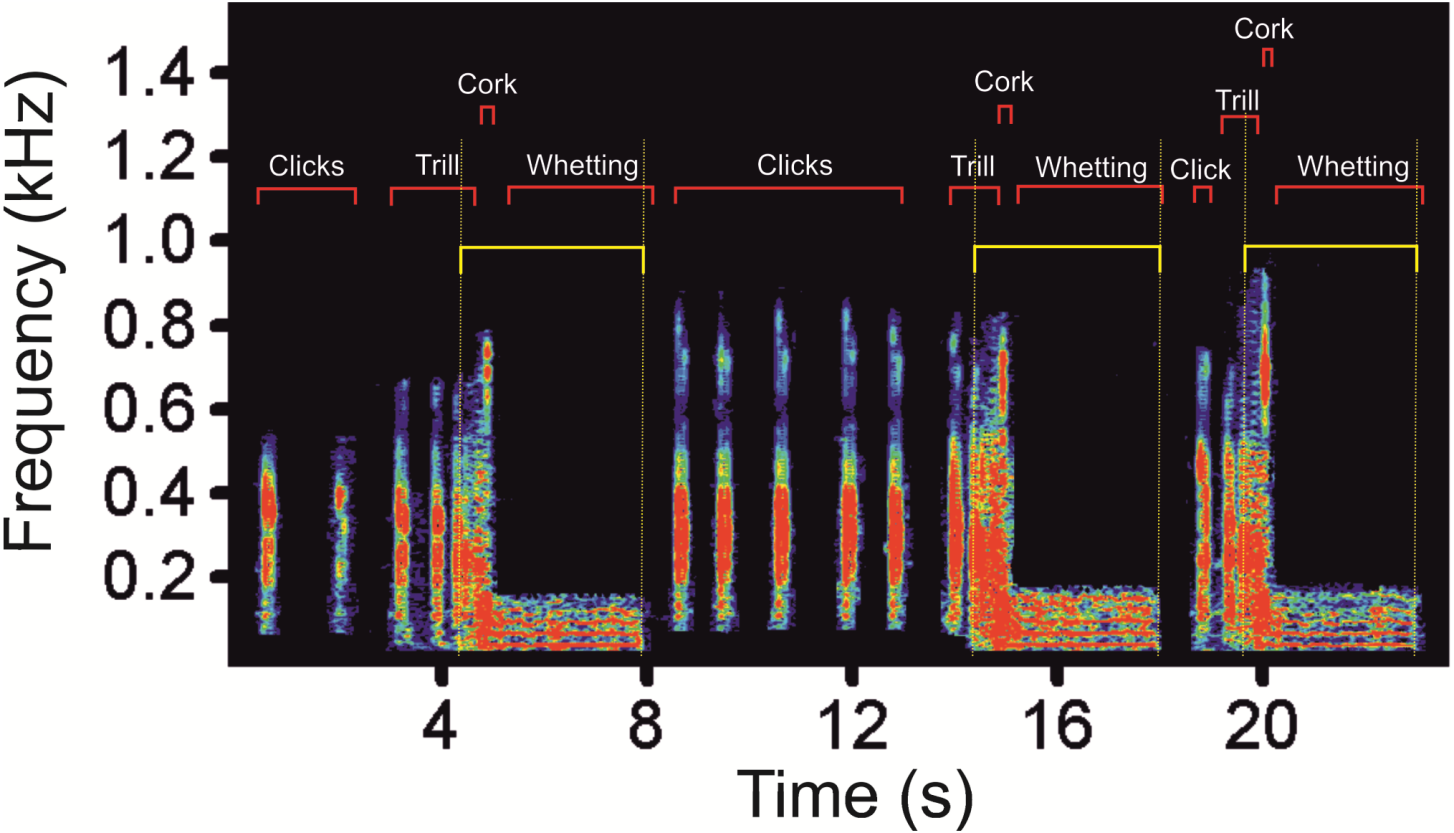
A spectrogram of the low-frequency part (as delimitated by horizontal yellow lines) of three subsequent display vocalizations of the Western Capercaillie.

**Figure 3:**
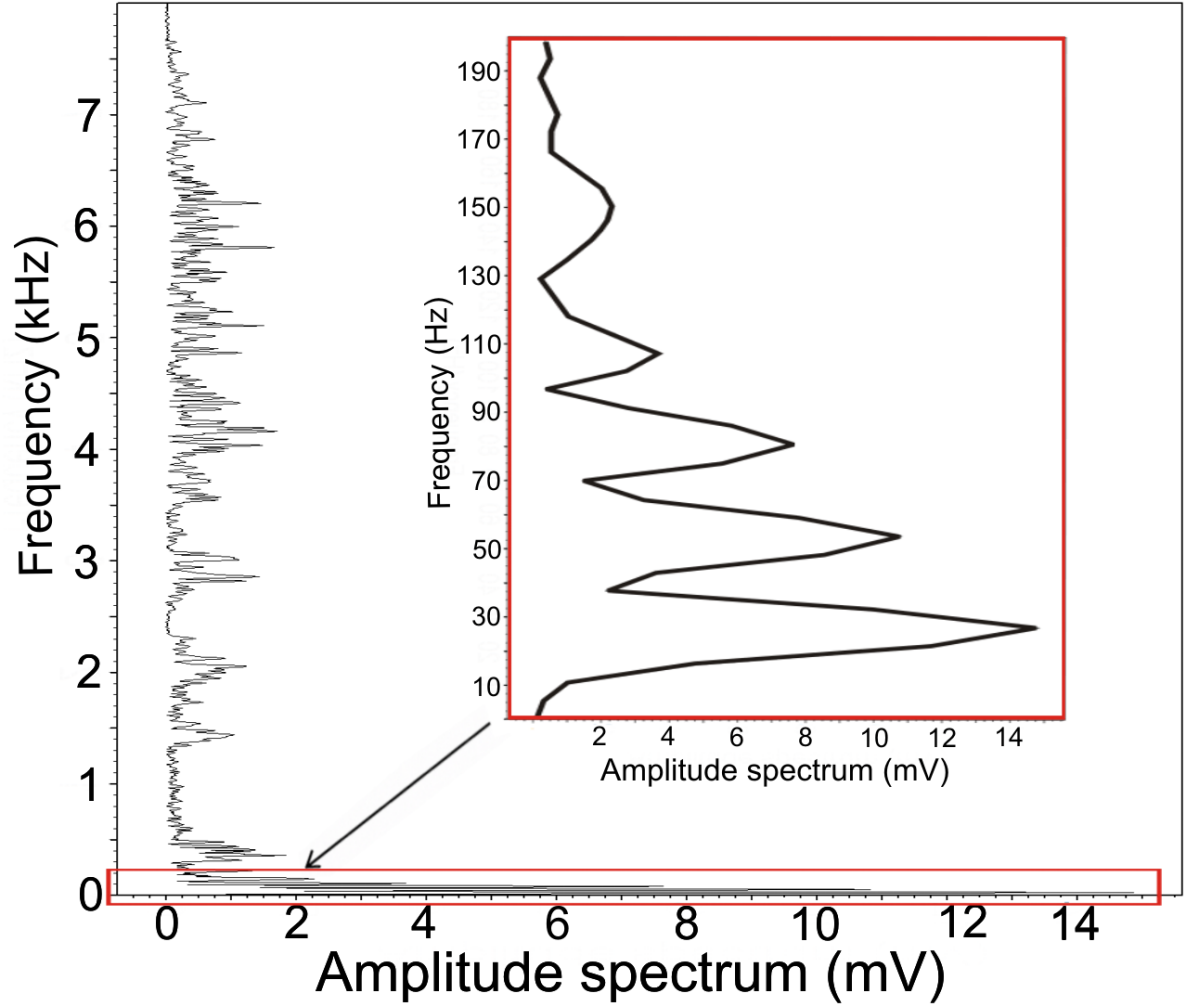
Amplitude spectrum of the low-frequency component. Increased frequency resolution (red rectangle) reveals a fundamental frequency of 27 Hz. This fundamental frequency also represents the most intensive frequency with three harmonic frequencies.

We found low-frequency harmonically structured signals with a fundamental frequency of 28.7 ± 1.2 Hz (Table 1) and two to five apparent harmonics (Fig. 2). The most intensive frequency was found on each of the first four harmonic tones, but most frequently on the second harmonics, as the peak frequency reached 63.5 ± 21.9 Hz and the duration of these signals was 3.13 ± 0.16 s. These low-frequency components mostly overlapped with the Whetting phase in 96% of its duration. Whetting represents the loudest phase of the display vocalization, thus masking the low frequency. Therefore it was necessary to increase frequency resolution of the lowest frequency band, i.e., the frequency range of 0–200 Hz (see Fig. 3) to reveal these components. A smaller part of low-frequency harmonics overlapped with the Trill phase (10.2%), and the Cork was always included. The Bonferroni-corrected Kruskal–Wallis test showed significant differences in all measured acoustic parameters: 16.76 >H (7, *N* = 108) <64.89; *p* < 0.002 (Table 1).

**Table 1:** Descriptive statistics and Kruskal Anova Test (SD) standard error of the mean. (Kruskal–Wallis test) Test of differences among individuals. (Bonferroni correction) *P* value adjusted acording Bonferroni correction.

Variable correction	Mean	Min	Max	SD	Kruskal–Wallis test H (7, *N* = 108)	*p*	Bonferroni
F0 (Hz)	28.69	27.0	32.0	1.23	56.47	*p* < 0.001	*p* < 0.001
Peak F (Hz)	63.46	27.0	94.0	21.87	56.00	*p* < 0.001	*p* < 0.001
Low F duration (s)	3.13	2.07	3.52	0.16	47.74	*p* < 0.001	*p* < 0.001
Song duration (s)	6.19	3.98	18.85	2.36	38.27	*p* < 0.001	*p* < 0.001
Trill duration (s)	1.25	0.48	2.33	0.39	32.96	*p* < 0.001	*p* < 0.001
Whetting duration (s)	2.85	2.37	3.28	0.15	64.89	*p* < 0.001	*p* < 0.001
LowF-Trill overlap (%)	28.44	9.04	77.4	11.91	43.88	*p* < 0.001	*p* < 0.001
LowF-Wheet overlap (%)	96.03	87.09	116.56	3.71	16.77	*p* < 0.019	*p* < 0.002

**Figure 4:**
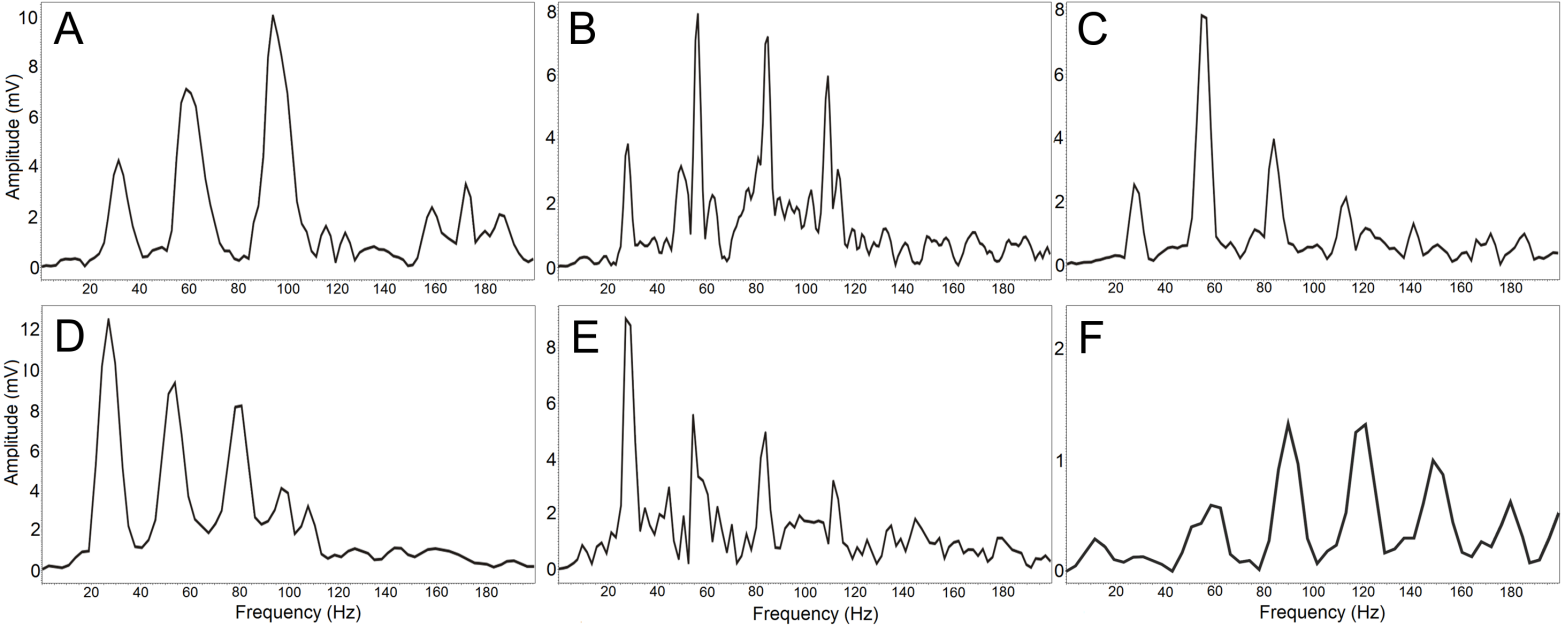
(A-F) The amplitude spectrum of the low-frequency components shows an individually distinct pattern in six males. The most intensive frequency may be found in each of the first four harmonic frequencies.

The resulting DFA model (Wilks’s Lambda = 0.091) included four significant discriminating variables (*p* < 0.001): PeakF, Whetting dur, LowF to Trill overlap, LowF to Whetting overlap. This discrimination model assigns 67.6% display vocalizations (63%, cross-validated result) correctly to the correct individual in comparison to a probability of 12.5% according to assignment by chance. The first discrimination function mostly correlated with PeakF (*r* =  − 0.56) and Whetting dur (*r* = 0.56). With the second discrimination function, LowF correlated to Trill overlap (*r* =  − 0.72). The first two discrimination functions described 87.1% of variation. The amplitude spectrum of the low-frequency components shows the individually distinct pattern (Fig. 4).

## Discussion

The frequency at 32 Hz in the southern cassowary (*Casuarius casuarius*) and 23 Hz in the dwarf cassowary (*Casuarius bennetti*) (Mack & Jones, 2003) were reported as the lowest frequency of vocal signals in birds. The occurrence of such low frequencies in the Western Capercaillie (minimum fundamental frequency 26 Hz) is therefore surprising because this grouse is substantially smaller (up to 6.5 kg) than cassowaries weighting up to 29 kg (Dwarf Cassowary) or even up to 58 kg (Southern Cassowary).

The conspicuous vocal display of the Western Capercaillie males indicates its significant function during sexual courtship, probably attracting females. Low frequencies are favourable for long-distance signalization as longer waves better propagate through vegetation (Heimann, 2003).

Another function of low-frequency components in the display vocalizations of mating males would be signalization of their size, which can indicate fighting ability (Clutton-Brock & Albon, 1979; Clutton-Brock, Hiraiwa-Hasegawa & Robertson, 1989). Low frequencies could thus not only be heard by hens over a long distance but also used for evaluating the male’s quality during mate choice. Individually distinct pattern of these low frequency components could indicate their influence for sexual selection. It is known that most females of the Western Capercaillie select the same dominant male (Wiley, 1974). On the other hand, we recorded birds from a short distance and thus we do not know what is happening to the signal during propagation over longer distances, when it actually reaches the female ear from a distance of dozens meters. It has been shown that individually distinct information can change during signal propagation to a receiver (Budka & Osiejuk, 2014). By contrast, interactions between males and females on the lek can take at short distance. Females could be attracted to lek by well heard frequencies of males’ mating calls or another long-distance signal (e.g., hissing call) from a longer distance. They move toward calling males on a lek so they usually can hear more than one male during searching mating. After approaching to calling males at close distance, females could make the choice decisions on the base of the ques reflecting the quality of male based on low-frequency components. It has been found that females in captivity preferred to mate with males with a longer calling activity (Rosenberger et al., 2020). The final female choice at a closer distance could reflect the tradeoff between finding a high-quality mate and predation risk including energetic costs from evaluation of several males on lek.

Although hearing in the Western Capercaillie has not been studied, the ability to hear these low frequencies is probable. Hearing of infrasound has been documented for homing pigeons (Heffner et al., 2013; Kreithen & Quine, 1979), the domestic chicken (Hill et al., 2014), and the guinea fowl (Theurich, Langner & Scheich, 1984). While a low frequency vocalization might be advantageous and increase the fitness of the male Western Capercaillie, it is apparent that the low-frequency phase correlates with the period of temporary deafness which is known only during the Whetting phase, which is used by hunters of this otherwise very shy bird (Bray & Thurlow, 1942). One of the mechanisms responsible for auditory self-masking is the contraction of the middle ear muscles (Borg & Counter, 1989; Borg & Zakrisson, 1973). We suggest that while it may be advantageous for communication and making an impression to vocalize in a very low frequency range, the Western Capercaillie pays with temporary deafness while singing - a deafness which made it also so popular (not only) among hunters.

Because these low-frequency components temporarily overlap with the Whetting phase, they are consequently hardly audible. It is possible to hear a growling resonating sound accompanying the whetting at a closer distance up to three meters. This fact is likely to cause a long time to escape attention, though the vocalization of the Capercaillie was studied.

Low frequencies could enable better information transfer and thus may be advantageous for long-distance signalization as longer waves propagate better through vegetation and thus enable improved information transfer.

## Conclusions

This experiment is the first time that such low frequencies were unambiguously proven in vocalization of birds smaller than cassowaries. We found low-frequency harmonically structured signals with a fundamental frequency and two to five apparent harmonics. Although hearing in the Western Capercaillie has not been studied, the ability to hear these low frequencies is probable. The conspicuous vocal display of males indicates a significant function during courtship interactions. Low frequencies may be advantageous for long-distance signalization as longer waves better propagate through vegetation and thus enable better information transfer. Information gained from the fundamental frequency contour could be the key parameter used for potential information about male identity during both male–female and male-male interactions. Indeed, we found a significant distinction of low-frequency signals among males.

##  Supplemental Information

10.7717/peerj.9189/supp-1 Supplemental Information 1 Measured acoustical parameters (ID) Identity corresponding to DFA results. (Local) Locality. (F0) Fundamental frequency. (Peak F) frequency of the highest intensity. (LowF) Duration of the low-frequency component. (Dur) Duration of the whole song. (Trill) Duration of the Trill phase.(Whet) Duration of the Whetting phase. (Low-Trill) Temporal overlaps of LowF duration and Trill duration. (Low-Whet) Temporal overlaps of LowF duration and Whetting duration.

## Additional Information and Declarations

### Data Availability

The following information was supplied regarding data availability: The measured acoustical parameters are available as a Supplementary Files.
