# Supplementary material for: Low frequencies in the display vocalization of the Western Capercaillie (Tetrao urogallus)
Source: PeerJ. 2020 Jul 8;8:e9189. doi: 10.7717/peerj.9189 (PMC7353911; doi:10.7717/peerj.9189)
Supplement: Supplemental Information 1 — (ID) Identity corresponding to DFA results. (Local) Locality. (F0) Fundamental frequency. (Peak F) frequency of the highest intensity. (LowF) Duration of the low-frequency component. (Dur) Duration of the whole song. (Trill) Duration of the Trill phase.(Whet) Duration of the Whetting phase. (Low-Trill) Temporal overlaps of LowF duration and Trill duration. (Low-Whet) Temporal overlaps of LowF duration and Whetting duration. [file peerj-08-9189-s001.docx]

ID Local F0 Peak F LowF Dur Trill Whet Low-Trill Low-Whet

1 BAV 31 92.0 3.04 6.217 1.50 2.82 20.49 94.17

1 BAV 31 92.0 2.88 6.890 1.46 2.80 10.67 94.25

1 BAV 32 90.0 2.97 10.959 1.73 2.82 14.05 94.21

1 BAV 31 93.7 2.94 14.837 1.98 2.81 10.01 94.87

1 BAV 29 90.0 3.04 5.642 1.73 2.82 16.43 95.20

1 BAV 29 90.0 3.04 4.870 1.25 2.82 22.36 95.20

1 BAV 29 78.0 3.00 5.323 1.11 2.81 21.45 95.55

1 BAV 29 82.0 3.00 7.093 1.92 2.82 12.13 95.56

1 BAV 28 86.0 3.10 8.173 1.54 2.84 20.14 95.56

1 BAV 32 93.7 3.04 5.363 1.17 2.82 24.27 94.82

1 BAV 30 78.0 3.07 6.246 1.63 2.84 16.13 95.84

1 BAV 30 90.0 3.04 5.960 1.25 2.86 21.04 94.40

1 BAV 29 90.0 3.04 7.290 1.73 2.82 16.47 94.51

1 BAV 28 86.0 3.04 4.885 1.17 2.84 23.61 94.33

2 SUM 28 80.0 3.20 4.545 1.08 2.64 24.47 107.67

2 SUM 29 85.9 3.07 8.829 1.83 2.86 13.90 94.29

2 SUM 30 90.4 2.91 6.431 1.56 2.65 17.81 95.32

2 SUM 28 58.7 2.84 4.748 0.75 2.56 31.83 97.89

2 SUM 28 62.8 2.94 4.911 0.83 2.65 26.27 99.28

2 SUM 29 86.0 2.97 8.707 2.33 2.66 9.04 100.30

2 SUM 30 90.0 3.36 5.394 1.41 3.07 23.06 94.93

2 SUM 30 29.4 3.29 6.349 1.75 2.86 28.52 93.91

2 SUM 32 94.0 3.23 5.589 1.78 2.88 23.25 94.27

2 SUM 30 86.2 3.13 5.674 1.30 2.85 28.37 93.33

3 SUM 30 86.0 3.16 7.253 2.05 2.83 18.12 95.34

3 SUM 28 82.2 3.13 4.877 1.27 2.85 26.71 95.02

3 SUM 30 52.8 3.07 6.024 1.51 2.83 18.23 94.81

3 SUM 28 82.2 3.07 5.386 1.64 2.86 16.26 94.34

3 SUM 28 81.3 3.16 6.048 1.63 2.89 20.60 95.02

3 SUM 28 82.6 3.16 4.984 1.36 2.80 22.98 98.78

3 SUM 28 54.8 3.10 5.288 1.45 2.85 20.34 94.73

3 SUM 28 82.2 3.04 5.074 1.34 2.84 18.42 94.51

3 SUM 28 80.6 3.10 5.624 1.92 2.90 14.72 94.72

3 SUM 30 85.1 3.26 5.173 1.31 2.87 32.16 95.69

3 SUM 28 57.0 3.23 4.578 1.05 2.82 29.38 99.54

3 SUM 28 80.2 3.10 5.645 1.83 2.87 17.50 93.82

3 SUM 28 56.8 3.07 5.322 1.57 2.87 19.30 93.38

4 SUM 28 54.8 3.10 4.626 0.74 2.84 44.85 94.69

4 SUM 28 56.8 3.07 4.858 0.84 2.86 29.81 95.06

4 SUM 28 56.8 3.26 4.890 0.87 2.88 50.35 95.00

4 SUM 28 84.6 3.04 6.408 1.26 2.87 16.93 95.29

4 SUM 28 59.2 3.20 4.818 0.48 2.95 67.43 94.61

4 SUM 29 86.0 3.29 6.034 0.92 3.14 37.84 91.31

4 SUM 28 58.8 3.52 6.165 1.02 3.03 51.95 96.13

4 SUM 30 59.0 3.48 4.861 0.83 2.87 77.44 95.65

4 SUM 30 56.8 2.94 4.952 0.96 2.65 31.04 96.27

4 SUM 30 56.8 3.10 4.986 0.95 2.85 28.54 93.54

4 SUM 30 57.0 3.04 6.096 0.93 2.91 28.12 92.58

4 SUM 30 57.0 3.16 5.549 1.19 2.87 28.89 95.26

4 SUM 30 56.8 3.16 6.504 1.82 2.84 18.26 95.91

4 SUM 30 56.8 2.94 5.688 1.32 2.72 22.25 93.53

4 SUM 28 56.8 3.10 4.736 0.75 3.00 39.41 90.44

4 SUM 29 56.8 3.13 5.149 0.98 2.88 32.62 94.17

4 SUM 28 80.2 3.07 5.114 1.05 2.90 25.10 93.35

5 SUM 27 28.0 3.13 7.357 1.04 2.98 23.14 95.30

5 SUM 27 27.0 3.13 5.928 0.66 2.97 41.45 94.34

5 SUM 27 27.6 3.20 5.690 1.03 2.94 33.33 95.34

5 SUM 27 80.2 3.13 5.317 0.81 2.97 34.81 94.70

5 SUM 27 27.4 3.23 14.664 1.65 2.99 20.85 94.79

5 SUM 27 56.8 3.23 6.616 0.64 3.26 40.03 90.01

5 SUM 27 57.0 3.16 14.007 0.95 3.28 28.09 87.09

5 SUM 28 27.4 3.13 4.266 0.73 2.97 38.48 94.37

5 SUM 28 27.4 3.16 10.226 1.58 2.97 16.48 95.72

5 SUM 27 54.8 3.23 5.338 0.91 2.97 34.54 95.82

5 SUM 27 27.4 3.26 5.349 0.93 3.01 33.41 96.34

5 SUM 27 55.7 3.20 8.000 1.16 3.00 22.46 95.97

5 SUM 27 27.6 3.20 5.738 1.01 3.01 28.27 95.24

5 SUM 27 27.6 3.16 10.994 1.97 2.99 13.26 95.16

5 SUM 28 56.8 3.23 6.541 1.44 3.01 19.62 95.92

5 SUM 29 57.0 3.20 18.850 1.83 3.01 14.90 95.71

5 SUM 27 27.4 3.16 7.413 2.14 2.96 12.00 95.37

5 SUM 28 57.0 3.23 8.549 1.59 3.01 19.17 95.61

5 SUM 28 28.0 3.20 7.674 1.85 2.99 14.36 96.02

5 SUM 27 56.8 3.26 6.178 0.72 3.14 47.98 91.14

5 SUM 29 29.6 3.23 5.770 1.30 2.96 25.58 96.01

5 SUM 27 27.4 3.26 5.880 1.22 2.97 29.44 96.09

6 POL 29 28.5 3.20 6.677 1.38 2.80 36.43 95.82

6 POL 29 28.3 3.52 6.072 1.30 2.79 53.11 98.49

6 POL 28 27.8 3.23 11.096 0.93 3.05 35.24 92.45

7 POL 28 27.6 3.07 5.320 1.01 2.82 30.75 94.75

7 POL 30 56.8 3.00 4.218 0.86 2.80 37.86 92.53

7 POL 30 58.8 3.13 6.082 1.25 2.74 34.29 95.37

7 POL 30 29.4 3.00 4.314 0.95 2.77 37.20 92.85

7 POL 30 58.8 3.04 4.424 1.01 2.79 35.82 93.12

7 POL 30 58.8 3.07 4.837 1.19 2.76 28.48 94.85

7 POL 30 29.4 2.07 6.600 1.15 2.51 39.08 98.09

7 POL 30 29.6 3.04 6.848 1.02 2.57 40.86 97.82

7 POL 30 87.9 3.00 4.373 0.97 2.54 29.84 102.37

7 POL 30 90.2 3.00 5.456 0.93 2.61 27.12 101.65

7 POL 28 84.2 3.10 4.869 1.10 2.80 37.25 92.04

7 POL 28 86.2 3.10 5.138 0.90 2.63 43.51 99.54

7 POL 30 58.8 3.13 4.656 1.09 2.63 35.58 100.80

7 POL 30 58.8 2.94 3.984 0.57 2.37 42.81 100.59

8 POL 28 76.6 3.39 5.544 1.31 2.46 20.17 116.56

8 POL 28 53.9 3.10 4.792 1.28 2.83 26.25 95.23

8 POL 28 51.0 3.26 5.040 0.86 2.62 47.62 102.06

8 POL 28 51.9 3.13 4.181 0.95 2.67 29.31 103.23

8 POL 28 78.4 3.32 4.349 0.96 2.83 30.52 103.79

8 POL 28 54.8 3.36 4.901 1.37 2.74 26.96 105.14

8 POL 28 82.4 3.29 5.309 1.35 3.06 23.92 93.82

8 POL 28 80.4 3.20 4.314 0.94 2.84 41.03 96.02

8 POL 28 82.0 3.20 4.648 1.08 2.89 35.12 94.61

8 POL 28 54.8 3.23 6.040 1.31 2.84 22.87 100.42

8 POL 28 55.0 3.26 5.154 0.98 2.83 37.42 99.75

8 POL 28 82.1 3.39 4.381 0.99 2.78 52.72 100.00

8 POL 28 84.1 3.00 5.869 1.98 2.79 13.26 95.66

8 POL 28 80.4 3.10 4.469 1.09 2.79 37.41 94.69

8 POL 29 84.2 3.13 4.925 1.06 2.66 28.48 103.24

Measured acoustical parameters.

(ID) Identity corresponding to DFA results. (Local) Locality. (F0) Fundamental frequency.

(Peak F) frequency of the highest intensity. (LowF) Duration of the low-frequency component. (Dur) Duration of the whole song. (Trill) Duration of the Trill phase.

(Whet) Duration of the Whetting phase. (Low-Trill) Temporal overlaps of LowF duration and Trill duration. (Low-Whet) Temporal overlaps of LowF duration and Whetting duration.
